# Acute Heart Failure Due to Aluminum Phosphide Poisoning

**DOI:** 10.14797/mdcvj.295

**Published:** 2021-09-09

**Authors:** Marija Petrovic, Diana Otero, Adam Leigh, Vikas Singh

**Affiliations:** 1 Department of Medicine, Icahn School of Medicine at Mount Sinai, New York, US; 2 Cardiovascular Medicine Department, University of Louisville School of Medicine, Louisville, Kentucky, US

**Keywords:** aluminum phosphide, poisoning, chest pain, heart failure

## Abstract

Aluminum phosphide (ALP) is a pesticide agent and infrequent culprit of accidental poisoning. We present a case of severe reversible cardiomyopathy and left ventricular apical thrombus in a patient who worked as an exterminator and had ALP poisoning.

## Introduction

Aluminum phosphide (ALP) is commonly a suicidal agent in some countries; however, accidental poisoning is rare.^1^ Myocardial damage occurs in 60% to 100% of cases of ALP intoxication. It can manifest as pericarditis or myocarditis, new-onset heart failure, subendocardial infarction, refractory hypotension, and shock. We present a case of severe reversible cardiomyopathy and left ventricular (LV) apical thrombus in an exterminator with ALP poisoning.

## Case Presentation

A 38-year-old male presented to the emergency department with persistent left-sided chest tightness and shortness of breath for 4 hours. He worked as an exterminator and his symptoms started 30 minutes after experiencing mask malfunction while fumigating a house. He smoked a half pack of cigarettes daily and denied illicit drug use or alcohol abuse; the rest of his past medical history was unremarkable. Vital signs showed a heart rate of 105 bpm and pulse oximetry of 92% on room air. He was tachypneic, had normal heart and lung sounds, and had no jugular vein distension or lower extremity edema. Arterial blood gas revealed a pH of 7.47, pCO2 36 mm Hg and pO2 59 mm Hg. Other laboratory testing was significant for serum troponin I of 1.6 ng/mL; aspartate transaminase and alanine transaminase 39 U/L and 43 U/L, respectively; BNP of 265 pg/mL; and lactic acid of 1.4 mg/dL. He tested negative for COVID-19.

**Figure 1:**
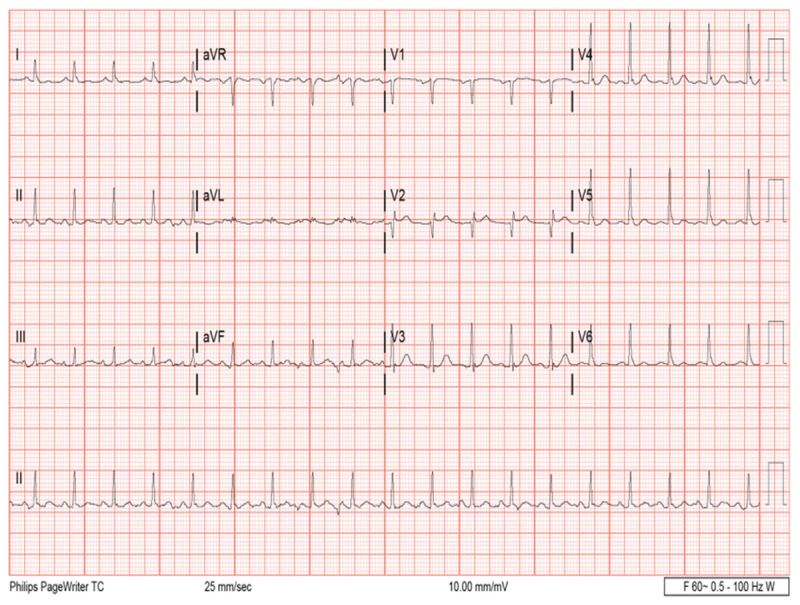
Electrocardiogram shows sinus tachycardia.

**Figure 2:**
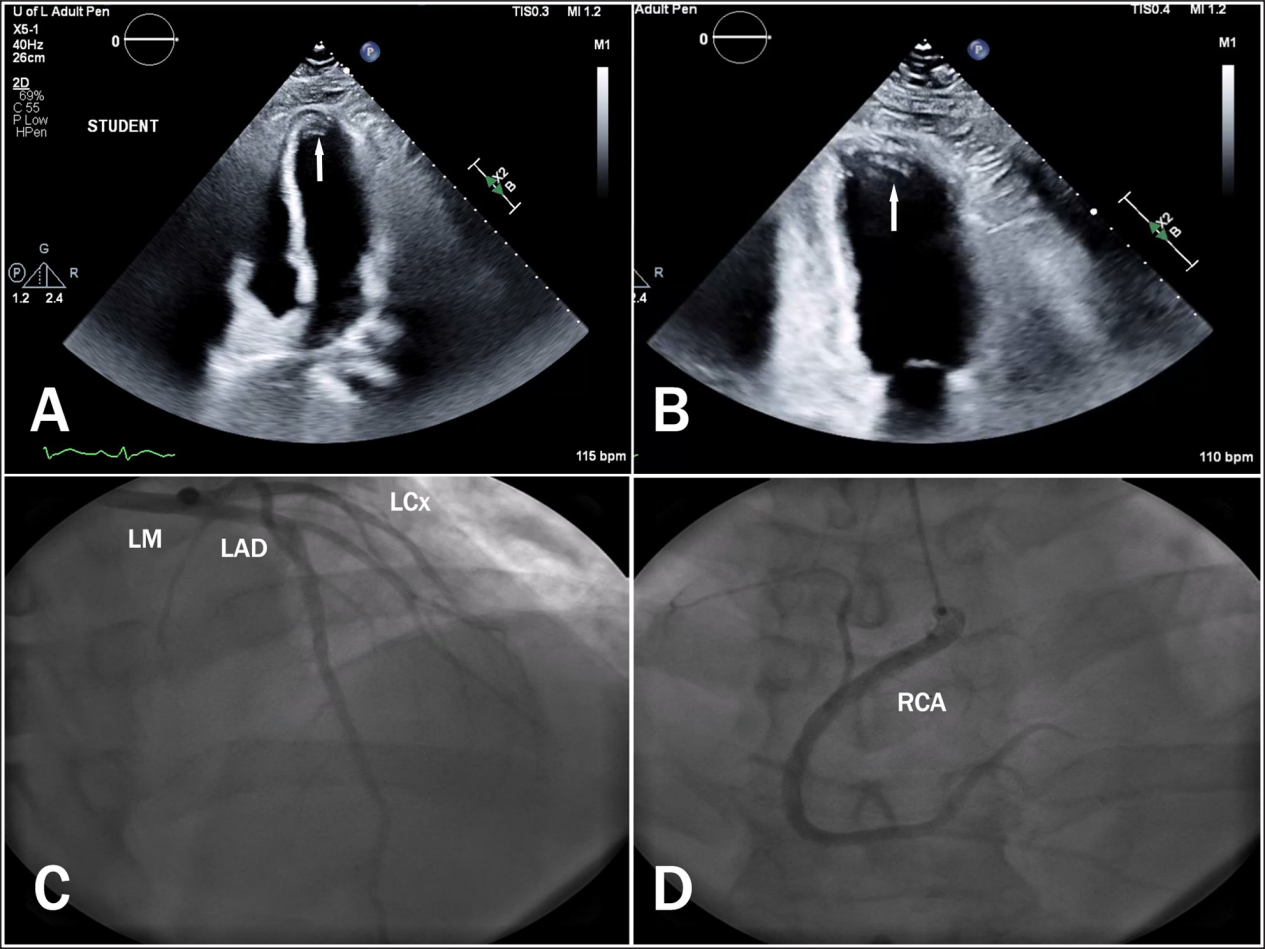
Initial transthoracic echocardiography shows severe global hypokinesis with apical immobile thrombus (arrow) in **(A)** apical 4-chamber and **(B)** apical 2-chamber views. Coronary angiogram shows normal epicardial arteries from **(C)** left anterior oblique and **(D)** right anterior oblique views. LM: left main coronary artery; LAD: left anterior descending artery; LCx: left circumflex artery; RCA: right coronary artery.

**Figure 3:**
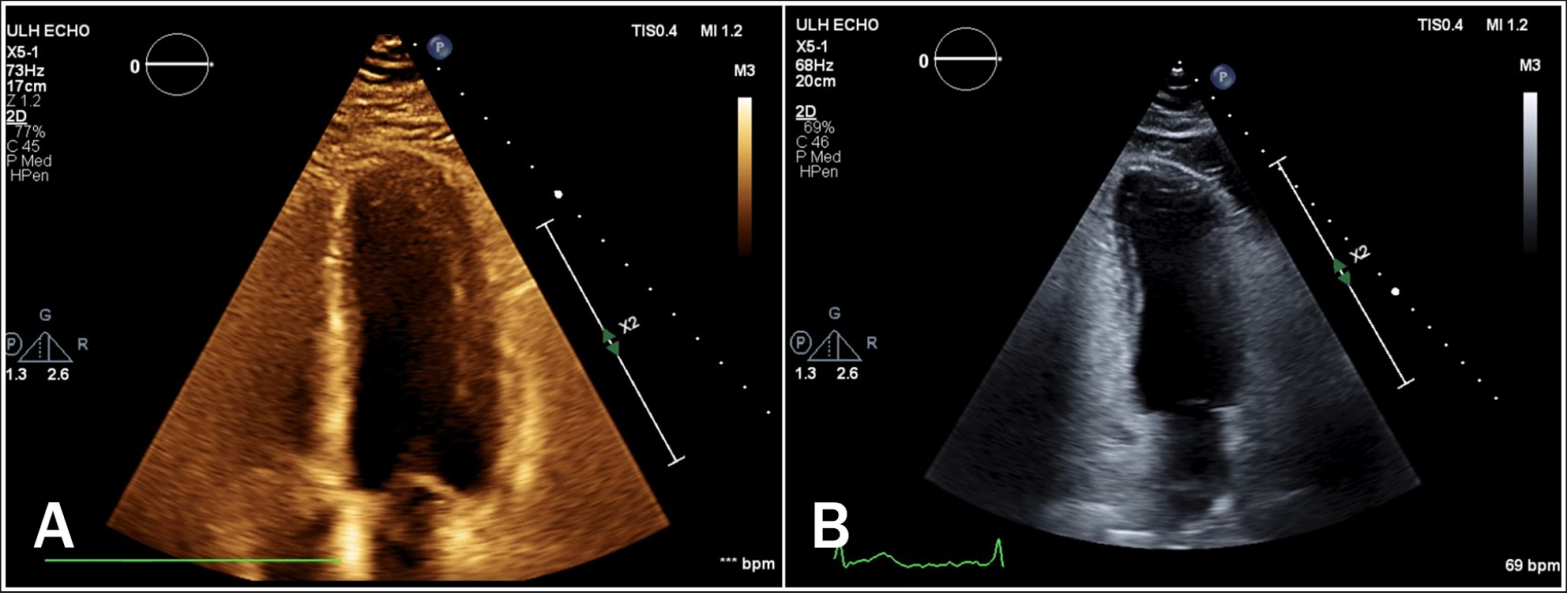
Follow-up transthoracic echocardiography with recovered left ventricular function in **(A)** apical 4-chamber and **(B)** apical 2-chamber views.

A chest x-ray revealed mild pulmonary congestion and an electrocardiogram confirmed sinus tachycardia (***Figure 1***). A TTE demonstrated severe global left ventricular dysfunction with a left ventricular ejection fraction (LVEF) of 20% and an immobile apical mural thrombus (***Figure 2A*** and ***2B***; ***Videos 1*** and ***2***). A coronary angiogram showed normal epicardial coronaries (***Figure 2C*** and ***2D***). The patient was diagnosed with ALP poisoning. He was managed with supportive treatment and started on goal-directed medical therapy for acute decompensated heart failure. He achieved full recovery, and TTE 1 month later revealed LVEF recovery to 60% without evidence of apical thrombus (***Figure 3A*** and ***3B***; ***Videos 3*** and ***4***). We continued treatment for 4 months and then stopped.

**Video 1:** Apical 4-chamber view of initial transthoracic echocardiography showing severe global hypokinesis with apical immobile thrombus. *https://youtu.be/1R-7bDu0tfk*

**Video 2:** Apical 2-chamber view of initial transthoracic echocardiography showing severe global hypokinesis with apical immobile thrombus. *https://youtu.be/GtvhKCqqyMM*

**Video 3:** Apical 4-chamber view of follow-up transthoracic echocardiography with recovered left ventricular function. *https://youtu.be/m-EywNUJ9aE*

**Video 4:** Apical 2-chamber view of follow-up transthoracic echocardiography with recovered left ventricular function. *https://youtu.be/-v0fw9MXO0E*

## Discussion

**Figure 4:**
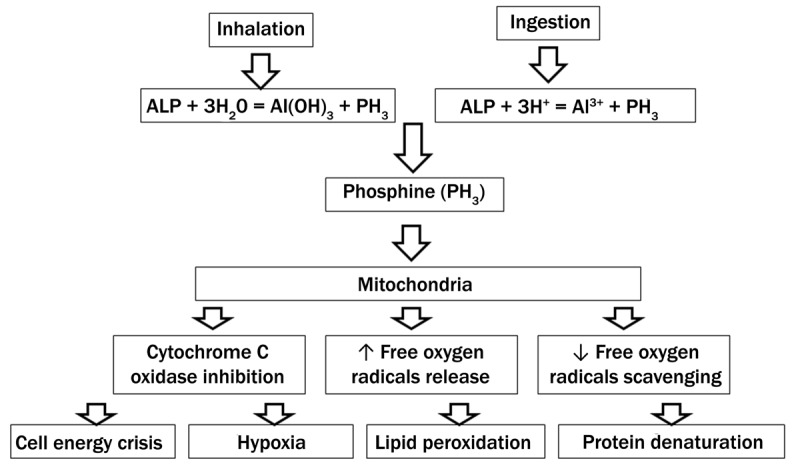
Mechanism of cellular injury caused by aluminum phosphide intoxication.

Inhaled or ingested ALP produces toxic phosphine when in contact with moisture. Phosphine noncompetitively inhibits cytochrome C oxidase and causes oxidative phosphorylation in mitochondria, leading to cell energy crisis and hypoxia. It also boosts mitochondrial release of free oxygen radicals that results in lipid peroxidation and protein denaturation of the cell membrane and inhibits the antioxidant enzymes, catalase and peroxidase, decreasing the scavenging of free radicals (***Figure 4***). The severity of intoxication depends on the dose ingested, and there is no specific antidote. Diagnosis is based on exposure history, gastric aspirate analysis, gas chromatography study/mass spectrometry method, or on the presence of specific phosphine odor (smell of garlic or decaying fish).^1,2^

The myocardial necrosis and changes in membrane action potentials cause nonspecific ST-T wave changes on electrocardiogram (EKG). EKG abnormalities and arrhythmias are a sign of a poor prognosis.^1^ Usual echocardiographic findings in ALP intoxication are decreased LVEF, generalized LV hypokinesia, and pericardial effusion. Management of ALP intoxication is largely supportive and has avery high risk of death, ranging from 37% to 100%.^1,2^

Our patient’s profession pointed to an occupational toxin exposure. Our initial differential diagnosis included acute coronary syndrome and myocarditis, but these were subsequently ruled out. We believe that the immobile apical thrombus in our patient formed because of blood stasis in the LV with severely depressed systolic function. A few similar cases were reported with complete reversibility of LV function in 1 to 2 weeks.^2^ Though the exact mechanism is still unclear, reperfusion-like injury with myocardial stunning due to cell energy crisis and oxidative stress may be proposed as the cause of reversible myocardial damage in phosphine poisoning.^3^ Inflammation (neutrophilic and eosinophilic infiltration), focal necrosis, myocytes vacuolation, and fibers fragmentation are typically found by autopsies.^4^

ALP is a widely used pesticide; however, the toxidrome may not always be easily recognizable by healthcare providers because intoxication may mimic other acute cardiac diseases. In our case, ALP-induced myocardial damage and dysfunction were reversed with supportive treatment and guideline-directed therapy for acute heart failure. However, specific guidelines for the management of phosphine cardiac toxicity and duration of the treatment are still lacking.

## Conclusion

Accidental ALP poisoning is rare. However, it is important to recognize it as a cause of acute cardiac failure in the appropriate patient. Furthermore, it is important to look early on for complications of severe heart failure, including ventricular thrombus formation.
